# Ten simple rules for being a co-author on a many-author non-empirical paper

**DOI:** 10.1371/journal.pcbi.1013317

**Published:** 2025-08-14

**Authors:** Friederike E. Kohrs, Natascha Drude, Anita Bandrowski, Tracey L. Weissgerber

**Affiliations:** 1 Berlin Institute of Health at Charité—Universitätsmedizin, QUEST Center for Responsible Research, Berlin, Germany; 2 Department of Neuroscience, University of California at San Diego—FAIR Data Informatics Laboratory, San Diego, California, United States of America; 3 SciCrunch Inc, San Diego, California, United States of America; 4 CNC-UC, Center for Neuroscience and Cell Biology, University of Coimbra, Coimbra, Portugal; 5 CIBB, Center for Innovative Biomedicine and Biotechnology, University of Coimbra, Coimbra, Portugal; Dassault Systemes BIOVIA, UNITED STATES OF AMERICA

## Abstract

Many-author non-empirical papers include “how to” articles, recommendations or consensus statements, roadmaps for future research, catalogs of ideas, or calls to action. These papers benefit the research community and broader academic ecosystem by addressing unmet needs or introducing new perspectives and approaches. Large, diverse authorship teams that examine an issue from many different perspectives can create valuable resources that individual co-authors could not develop independently, or in smaller groups. Realizing the potential of many-author non-empirical papers, however, requires very different strategies than researchers would typically use to write papers with fewer authors. In our process, a core team of lead writers typically works together to lead the content generation and writing processes, while many co-authors collaboratively create content and provide feedback on outlines and drafts. Challenges for co-authors may include learning to write a different type of paper, adapting to high-volume feedback, and understanding the very diverse perspectives shared by fellow co-authors. This paper outlines ten simple rules for being a co-author on a many-author non-empirical paper. Although the rules were developed for papers with at least 30 authors, some rules may be useful for many-author research papers or for non-empirical papers with fewer authors. Co-authors may also want to consult our companion paper on ten simple rules for leading a many-author non-empirical paper, as understanding the challenges faced by lead writers will help co-authors to contribute more efficiently and effectively.

## Introduction

**Fig 1:**
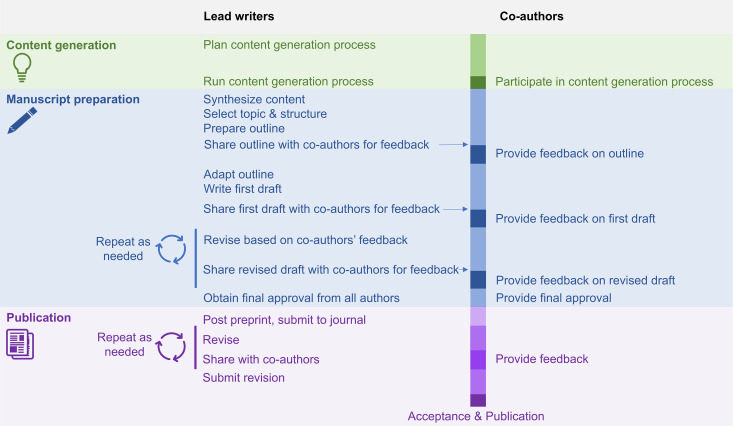
The process of preparing a many-author non-empirical paper. The infographic illustrates the activities of lead writers and co-authors during the three phases of completing a many-author non-empirical paper—content generation, manuscript preparation, and publication. Lead writers are typically highly involved in organizing the content generation process and writing the paper, while co-authors participate in content generation and provide feedback on the manuscript at specific points during the process. The general process shown here may be adapted based on the needs of the project or project team. Figure reprinted from [1].

Many-author non-empirical papers bring together large teams of experts to explore topics that are important to a research field, the entire research community, or to the public. These papers may create a new resource or expand the research community’s understanding of a topic by creating a collaborative synthesis that the individual authors would not have developed independently. Many-author non-empirical papers do not simply combine sections of text written by individuals, describing their own work, into a single paper. The process is typically highly interactive, with an intense content generation phase followed by a writing phase that includes multiple feedback rounds. Here we describe a workflow for content generation and manuscript preparation (Fig 1). While the number of authors needed for a many-author paper is not defined, the rules described in this article were developed while preparing papers with at least 30 highly engaged authors. Some rules may be useful for papers with fewer authors. Non-empirical here indicates that the paper is not original research, and does not follow the Introduction-Methods-Results-Discussion, or IMRD, format. Many-author non-empirical papers can have different formats, depending on the authors’ goals and journal formatting requirements. Options include recommendations or guidelines, consensus statements, roadmaps for future research, calls to action, or “how to” articles (Table 1).

**Table 1:** Types of many-author non-empirical papers.

Paper type	Goals and examples
Recommendations or consensus statements	Recommendations for achieving a particular goal [2]^*^
Catalog of ideas	Shares many ideas or approaches for achieving a particular goal, and may also briefly describe examples or cite resources for implementing each idea [3]
Call to action	Encourages stakeholders to take action on a specific topic, and outlines why this action is needed [4]
Roadmap for future research	Identifies key unsolved problems that are crucial for the advancement of a particular field
“How to” papers	Shares expert knowledge on how to do something, by providing practical input and examples [5]
Community resource	Creates a resource that others can use and build upon
Combination papers	Combines two or more of the categories above (e.g., [6] is a recommendation, roadmap, and a “how to” paper)

* Recommendations presented in non-empirical papers are generally developed through expert consensus, rather than systematic approaches, such as the Delphi processes [7]. Table reprinted from [1]. While there are many examples of these types of works, we have only cited works in which the authors of these ten simple rules papers participated. Other groups may have different content generation and writing processes, and we do not want to imply that they used the processes outlined in this paper.

Several factors make many-author non-empirical papers challenging to write. First, deciding on content and structure can be quite difficult. When writing an original research paper, the content is the authors’ research, and papers follow the IMRD structure. In contrast, the authors of a many-author non-empirical paper could potentially write many different papers, using different structures. Authors must make many decisions about which paper to write, what content to prioritize, and how to structure the paper to clearly convey the desired message(s). Second, researchers often have limited experience writing or critiquing non-empirical papers that aren’t narrative reviews, editorials, or commentaries. For many co-authors, this may be their first time writing a recommendation or consensus statement, roadmap for future research, call to action, or “how to” article. This “learning while doing” approach can affect feedback quality. Third, all contributors must adapt to accommodate high-volume feedback, while acknowledging their many co-authors’ diverse perspectives.

Despite these challenges, many-author non-empirical papers have several advantages. Large, diverse teams of expert contributors lead to more comprehensive and nuanced perspectives than one might achieve with a smaller team. The resulting papers resonate with many different audiences, leading to broader dissemination and applicability. The networks and connections formed while preparing a many-author non-empirical paper can lead to lasting collaborations or serve as a foundation for future actions. Co-authors may increase their reach and visibility while also quickly gaining a comprehensive overview of perspectives and latest developments in the field. All of these factors could potentially increase impact and are especially relevant for ECRs.

Many-author non-empirical papers are often led by a small team that designs and runs the content generation process and does most of the consolidation and writing. We will refer to these individuals as the lead writers. The authorship team also includes a large group of co-authors, who participate in the content development and provide feedback on the paper outline and drafts at several defined timepoints throughout the process. The experience of the lead writers is often very different from that of co-authors. Leading a many-author non-empirical paper is an intensive time commitment, and responsibilities include managing the complex logistics of content generation and manuscript preparation, making decisions about the scope, content, and structure, incorporating feedback from many co-authors, and resolving conflicts. Lead writers handle many situations behind the scenes that are not apparent to co-authors. In this paper, we provide ten simple rules for being a co-author on a many-author non-empirical paper (Table 2). We encourage readers to consult the companion article on “Ten simple rules for leading a many-author non-empirical paper” [1], as understanding the lead writers’ challenges will help you to contribute effectively as a co-author.

**Table 2:** Ten simple rules for being a co-author on a many-author non-empirical paper.

Number	Rule
**Rule 1**	Actively participate in content generation activities
**Rule 2**	Adapt your understanding of authorship
**Rule 3**	Provide timely feedback at all stages
**Rule 4**	Provide major feedback early. Address minor points later.
**Rule 5**	Be open-minded and comment constructively
**Rule 6**	If you’re feeling overwhelmed, focus on sections of the paper that are relevant to your expertise
**Rule 7**	Avoid adding text or self-citations purely to promote your work
**Rule 8**	Share comments within the draft, instead of emailing the lead writers
**Rule 9**	Practice good author hygiene
**Rule 10**	Remember the “golden rule” of many-author papers

### Rule 1: Actively participate in content generation activities

Many-author non-empirical papers typically begin with a content generation phase. This might be an unconference, a symposium, workshop, or a series of meetings (Box 1). Sometimes these events are specifically designed to lead to a paper. The content and structure of the planned paper may or may not be clearly defined. In other cases, an idea for a paper may emerge naturally during the course of the event. Ideally, the content generation activities foster interactive discussion and collaborative synthesis. Events consisting of a series of lectures, followed by a few questions, limit the teams’ ability to reach a shared understanding.

If the organizing team did not clearly define the topic and structure of the paper prior to the content generation activities, it’s likely that the extensive conversations that take place during the event could lead to many different papers. The strength of many-author non-empirical papers lies in the collaborative synthesis of ideas and experiences from a very diverse group of experts. Therefore, the organizers will likely choose a topic that is broad enough to appeal to many participants, while filling a recognized gap. This may include creating a resource that others can use or expanding the research community’s understanding of a topic.

Participating in or actively creating content generation activities provides a great opportunity, especially for early career researchers (ECRs), to broaden their network, identify potential collaborators, join communities, and increase their visibility. The content generation phase is also your opportunity to influence the scope, topics, and main messages of a future paper. Many-author non-empirical papers are often broad in scope, so use the content generation phase to understand how your interests fit within the broad range of topics that are discussed. When sharing your work, focus on building connections between your interests and those of other participants. Provide links to resources for participants who want to learn more. If the scope and content of the paper have not yet been determined, you may want to highlight knowledge gaps or other opportunities for the field to progress.

Content generation events are most effective when the organizers and participants create an inclusive space, where everyone can safely contribute. Due to the non-hierarchical and less structured nature of these non-traditional activities (Box 1), many participants are often ECRs. Compared to senior scientists, ECRs are more diverse and more likely to come from historically marginalized groups [8]. Participants should work to maintain an inclusive space, through actions such as adhering to codes of conduct, being respectful of differing experiences, ideas and perspectives, and leaving room for intellectual disagreement. This is particularly important for participants in positions of power, due to factors such as seniority or involvement in organizing and running the event. The approaches and behaviors used to create a safe and inclusive space should extend throughout the project, including the content generation, writing, revision, and publication phases.

**Box 1. Content generation and writing processes for many-author non-empirical papers.**

A few examples of content generation strategies that the authors have successfully used to write many-author non-empirical papers are listed below; however, this is not an exhaustive list. Lead writers can explore other formats, such as BarCamps [9] or World Cafés [10], or adapt existing formats to suit their needs. Box reprinted from [1].

***Unconferences:*** Unconferences, or unconventional conferences, seek to maximize the discussions and networking that occur during coffee breaks at traditional conferences. There are many different unconference formats, for in-person and virtual meetings. Examples include structured conversations (see the “Participant led workshops at the Future of Research Symposium” section of [11]) and virtual brainstorming events [12,13]. Virtual brainstorming events bring together researchers and other stakeholders for two days of asynchronous discussion on a specified topic [12,13]. The two-day events include virtual networking events, where participants get to know each other, webinars, where participants give lightning talks, virtual synchronous meetings for small group discussions on important topics, and lively asynchronous discussions on an online discussion board (e.g., Slack). Other ideas for starting focused conversations on topics relevant to the theme of an intended paper might include asking participants to discuss controversial statements or PechaKucha talks [14], comprised of 20 slides each shared for 20 seconds, where participants share a specific idea or experience. The unconference format was developed for global teams (e.g., [2,5]); however, it can also be used nationally (e.g., [3]) or locally.

***Meetings:*** Lead writers may organize a series of in-person or virtual meetings, where contributors share expertise and experiences. A moderator guides the discussion. Small groups of 4–6 people allow everyone to contribute. Organizers may either hold a series of small group meetings, or use breakout groups or rooms to facilitate small-group discussions, before bringing everyone together to share insights gained from each group.

***Intensive workshops:*** Organizers establish guiding questions or themes independently, or in consultation with workshop participants. The workshop itself may combine small group discussions, where participants explore particular questions in depth, and large group discussions, to synthesize and share information among all participants. Conversations are documented by notetakers or by using interactive tools or online whiteboards to complement brainstorming and discussion exercises.

***Writeathons and writing sprints:*** Lead writers may organize one or more collaborative, time-limited events to bring together various contributors, who work on achieving predefined content generation goals. Outputs for a session may include drafts of chapters or paper sections, or illustration mock-ups. Writeathons or writing sprints can facilitate rapid collaboration and content generation.

### Rule 2: Adapt your understanding of authorship

For most co-authors, participating in content generation is the intellectual contribution to authorship, fulfilling the “Investigation” role in the CRediT taxonomy. Co-authors will also typically fulfill the “writing—revising and editing” CRediT role by providing feedback on the outline and/or manuscript drafts (https://credit.niso.org/). Some participants in the content generation phase may choose not to be listed as authors on the paper. Common reasons include concerns that they haven’t participated enough to merit authorship or a lack of time to provide feedback. Occasionally, a participant may choose not to be an author if some content in the paper doesn’t align with their views.

Resist the urge to keep score or make many minor comments to ensure that your contribution is visible. If you are the 30^th^ author to review the draft, others have probably already commented on the points that you would like to see addressed. When you agree with a comment, simply add a “+1” or a thumbs up. If you disagree with a comment that someone else has posted, politely explain why. Avoid intensely scrutinizing the draft for very minor points to make your contribution visible. If everyone does this (see Rule 10), the lead writers may need to sift through hundreds of very minor comments to find major comments that will affect the paper’s scope, structure, or content.

Author order may not be particularly meaningful on a many-author paper, as it’s very difficult to quantify co-author contributions. Contributions during content generation are difficult to quantify, as participation differs among individuals and co-authors share many interesting ideas that aren’t ultimately included in the paper. Furthermore, co-authors who read the draft later in the commenting period typically have fewer comments, as major points have already been identified by others. Hence, the number of comments that a co-author makes is not meaningful. Authorship roles may also shift and evolve over time. Passionate individuals, who become strongly involved in the content generation and/or writing and revision process, may have additional roles that were not anticipated during content generation.

Lead writers will often be listed in the beginning and end positions, with co-authors listed as middle authors. The order of middle authors may be randomly assigned or alphabetical. Alternatively, some groups may use consortium or group authorships, by naming lead writers in the authorship list, and including co-authors in the writing consortium.

Please be aware that while participating in a many-author paper will broaden your network, it may have implications for peer review of future grants. Some funders do not allow co-authors to peer review funding applications. These policies typically do not recognize that co-authors on many-author papers often have limited interactions, compared to co-authors on papers with fewer authors. Potential co-authors may wish to examine policies for funding agencies that they apply to regularly.

### Rule 3: Provide timely feedback at all stages

When sharing outlines or drafts, the lead writers will typically ask you to respond within a designated time frame. Whenever possible, provide feedback at each stage of the process, within the requested time frame. If you are unable to provide feedback at all phases, prioritize commenting on the outline and first draft. Major changes to manuscript scope, structure, and content are most likely to occur during these early phases, whereas changes on revised drafts are typically minor adjustments to content or phrasing. Co-authors who request major changes to the scope, structure, and content on revised drafts may find that these changes are no longer possible, as the co-author team has already reached consensus (see Rule 4).

If you miss a feedback round, carefully review others’ comments and the explanation of changes provided by the lead writers before examining the next draft to avoid repeating feedback that was given earlier. Pay special attention to requests that were not implemented and the rationale for these decisions.

### Rule 4: Provide major feedback early. Address minor points later

When reviewing the outline and first draft, comment on any major concerns that may affect the paper’s scope, structure, and content. These changes are easiest to make at the outline and first draft stages, while co-authors are building consensus about the paper’s main message(s). Co-authors who comment extensively on minor issues, such as adjusting phrasing or adding nuances to specific points, may waste time if those sections are later cut. As discussed in Rule 2, extensive copyediting or many minor comments on early drafts make it difficult for the writing leads to find major comments that should be addressed early.

When reviewing revised drafts, focus on nuances, phrasing, or other minor adjustments to improve clarity and refine the message. You may also suggest citations for key points, correct typos, or help with any other tasks where the lead writers have requested support.

Draft your comments so that lead writers and co-authors can easily understand the change that you’re requesting and how it is relevant to the paper’s main message(s). Co-authors differ in their expertise, experiences, and epistemological background. Terms and concepts may also differ across fields and cultures, and things that are obvious to you may not be obvious to others. Be especially careful when using abbreviations that are obvious in your field, as the same abbreviations can mean very different things in different fields. If possible, eliminate abbreviations or uncommon idiomatic phrases. Include references or examples to explain major comments. Vague comments without examples or citable references make it difficult for the lead writers to decide whether the comment is relevant. Make sure that your real name appears on each comment so that the lead writers can ask you if they have questions.

### Rule 5: Be open-minded and comment constructively

Large author groups include individuals with many different backgrounds and perspectives. Authors will not have the same views and opinions. Be aware of your own positionality and intentionally reflect on your assumptions and viewpoints [15]. Encourage others to do the same. Think about how your comments or replies might be perceived by others. Take some “cooling off” time before posting on views or aspects with which you strongly disagree. Framing comments constructively, instead of harshly criticizing the manuscript, is more informative for everyone. This might include acknowledging when your opinions, or those of others, may be controversial, or suggesting that the lead writers acknowledge the range of diverse perspectives, instead of presenting something that may be controversial as a consensus opinion. Leaving space for intellectual disagreement can help readers understand different positionalities and perspectives within one field.

Keep in mind that within a large author group, authors may use different terms to describe similar concepts. Explaining concepts in a way that is clear to readers with different backgrounds is especially important. When addressing controversial issues, suggest alternate wordings and explain your suggestions in a comment instead of changing wording directly in the draft. This prevents “editing wars” within the draft and makes it easier for co-authors to collaboratively refine the proposed wording to reach a consensus.

### Rule 6: If you’re feeling overwhelmed, focus on sections of the paper that are relevant to your expertise

The strength of many-author non-empirical papers is that they bring together co-authors with many different experiences, perspectives, and knowledge. The scope of these papers can be very broad. If you’re feeling overwhelmed, start by reading the entire paper to understand the scope and structure, and how the different topics fit together. Next, focus your feedback on the sections that are most relevant to your expertise. Other authors will provide feedback on sections where they are most knowledgeable.

When focusing on specific sections, always consider the balance between the section that you are editing and other sections before commenting. Many-author non-empirical papers contain many sections that could be expanded into independent papers. Some co-authors will want to add extensive details and nuances about topics related to their expertise. Co-authors are particularly likely to add content to sections on topics that are already widely studied or discussed, and this content often duplicates existing materials. Instead of adding extensive details on a widely discussed topic, add a sentence referring readers to other comprehensive resources and suggest appropriate citations.

### Rule 7: Avoid adding text or self-citations purely to promote your work

Only add things that are crucial to the main message(s) of the paper. Many-author papers typically get much longer each time that they are opened for comments, and need to be made more concise each time that they are closed for revision. We call this phenomenon “the accordion effect.” This occurs because most co-authors will add things, but very few will comment on things that could be removed.

Co-authors on many-author papers often conduct research or share resources that are linked to the topic of the many-author paper. In some cases, a co-author’s work is directly relevant and citing this work strengthens the paper by providing valuable context, filling a content gap, or providing an example of a phenomenon discussed in the paper. In other cases, many co-authors may have published papers or shared resources that are relevant to the topic mentioned in the paper. Lead writers and co-authors may prefer to cite one or two resources (e.g., the most comprehensive, those that provide the best example of the phenomenon described in the paper), instead of citing everyone’s work. Alternatively, the proposed resource may be outside the scope of the paper and divert readers’ attention from the paper’s main message(s).

When suggesting text or citations of your own work, ensure that the content is crucial to strengthen the main message of the paper. If you are unsure whether a description of your work fits within the scope of the paper, leave a brief comment explaining what text or citations you might like to add and why. This is more efficient, as lead writers can contact you to request additional text, and provide details about the desired length and main points.

### Rule 8: Share comments within the draft, instead of emailing the lead writers

Sharing your comments in the draft is crucial to ensure that your ideas are part of the evolving conversation among co-authors. Sometimes co-authors email the lead writers when they have particularly strong opinions, major concerns, or wish to see their own work cited. Occasionally, these emails contain sensitive information that the sender does not want to share with others. More frequently, however, these emails are intended to draw the writing leads attention to something that the co-author is passionate about. Private emails create two challenges for lead writers. First, comments that are only shared with the lead writers are not integrated into the conversation taking place in the draft, where co-authors are building consensus and identifying points of contention. Other co-authors can’t respond to opinions that aren’t shared publicly. This leaves lead writers in the challenging position of making an independent decision, or finding a way to introduce the ideas that were shared privately into the draft for broader discussion. Second, responding to emails from many co-authors, at each stage of the writing process, is very time consuming (see Rule 10). The emails are often long and detailed, and lead writers feel obligated to respond because they want to build consensus among co-authors.

### Rule 9: Practice good author hygiene

When starting to work on the outline or first version of the manuscript, provide essential information that the lead writers request from each author. Add your full name, correct affiliation, and ORCID, as well as your conflict of interest statement to the manuscript as soon as this information is requested. If your affiliation changes or if you need to add an affiliation, notify the lead authors as soon as possible. The affiliation in the paper should reflect where the work was done; specifically, your affiliation at the time of the content generation and feedback process. If multiple authors from your department or institution are involved in the same many-author paper, make sure that your affiliations are consistent. Familiarize yourself with your institutional conflict of interest regulations so that your conflict of interest statement is accurate and complete. Also update the lead authors if your email changes. Writing and publishing a many-author non-empirical paper can be a lengthy process, and lead writers will need to contact you throughout this process. You are responsible for ensuring that your information is correct and complete.

Once the manuscript has been finalized and is ready for journal submission, respond to final approval emails in a timely manner and make sure that all your information is entered correctly. Once submitted, link your ORCID in the manuscript submission system promptly and complete any required authorship forms from the journal to facilitate a smooth submission and review process. If the lead writers share page proofs, check the proofs carefully to ensure that your name, affiliation, ORCID, and conflict of interest statement are accurate.

### Rule 10: Remember the “golden rule” of many-author papers

As you provide feedback and await updates from the lead writers, you may find yourself wondering whether you should take a particular action. We encourage co-authors to imagine what would happen if all of your co-authors, or even 25% of them, were to do the same thing. Lead writers struggle to manage the volume of comments and emails that they receive from co-authors. Actions like emailing the writing leads to ask when the revised draft will be available, requesting another week to review the draft, or citing a few of your papers, may seem innocuous. Lead writers, however, spend a lot of time responding to these small requests, which add up when multiplied by many authors, throughout the content generation and writing process. These competing demands slow down revisions, delaying the process for everyone. Remember that each author is providing feedback and potentially contacting the lead writers during each phase of the process. This will help you to decide whether a request or action is crucial to the structure and content of the manuscript, or might have unintended consequences.

### Limitations and scope

This paper highlights one strategy that we have successfully used for writing many-author non-empirical papers, however, other groups may use different processes. This may include workflows in which co-authors are separated into writing groups to prepare specific sections, receiving feedback from a team of lead writers. Alternatively, groups may use processes in which the authorship team expands or contracts as needed. Additional lead writers may be added to write sections in which they are particularly knowledgeable, or lead writers may need to step back to devote time to other projects. Regardless of which process is used, having a core team that drives the project forward is crucial; otherwise, the paper is unlikely to be completed due to diffusion of responsibility and competing priorities. When merging sections from different authors or teams, substantial editing and revising is often needed to ensure that the manuscript is cohesive and has a clear line of argumentation. Designating a small group who is responsible for this revision process may lead to more cohesive results.

## Conclusion

Many-author non-empirical papers offer a unique opportunity to create valuable resources that no individual co-author could create independently. These papers have great potential to change the conversation or address unmet needs within research communities. Collaborating on a many-author non-empirical paper, however, is quite different from collaborating on a paper with fewer authors. Co-authors can enhance the writing process by adapting their approach to accommodate many diverse perspectives and high-volume feedback. Specific actions include adapting your understanding of authorship, prioritizing major comments that affect the content or structure of the paper, and providing timely and constructive feedback. As big team science becomes more common, researchers will increasingly need skills for collaborating on many-author non-empirical papers with a multidisciplinary and diverse authorship team. Developing these skills early in your career will help you to collaborate more efficiently and effectively, and can help you prepare to lead a many-author non-empirical paper in the future.
